# Therapeutic Effect of Intense Pulsed Light (IPL) Combined with Meibomian Gland Expression (MGX) on Meibomian Gland Dysfunction (MGD)

**DOI:** 10.1155/2020/3684963

**Published:** 2020-04-13

**Authors:** Shanshan Wei, Xiaotong Ren, Yuexin Wang, Yilin Chou, Xuemin Li

**Affiliations:** Department of Ophthalmology, Beijing Key Laboratory of Restoration of Damaged Ocular Nerves, Peking University Third Hospital, Beijing 100191, China

## Abstract

**Purpose:**

Our study aimed to evaluate the efficiency of intense pulsed light (IPL) combined with meibomian gland expression (MGX) in treating meibomian gland dysfunction (MGD).

**Methods:**

This study was a prospective interventional study. A total of 53 patients were included in the study and received a series of three treatments at an interval of 3-4 weeks. Follow-up examinations were completed 4 weeks after the last treatment. The Ocular Surface Disease Index (OSDI) questionnaire, tear meniscus height (TMH), tear break-up time (TBUT), slit-lamp examinations, and in vivo confocal microscopy (IVCM) were recorded before and after treatment. Additionally, an artificial intelligence automated software program was applied in our study for corneal nerve analysis.

**Results:**

The OSDI score was significantly reduced after the IPL treatment compared with baseline (*P* < 0.001). Meibomian gland assessment scores, including meibum quality and expressibility, eyelid margin abnormalities, and corneal staining, significantly decreased after treatment (*P* < 0.05). Moreover, the corneal nerve fiber length (CNFL) significantly increased after the treatment (*P* < 0.001).

**Conclusion:**

Intense pulsed light (IPL) combined with MGX is an effective treatment for MGD, and neurotrophism could be one of the mechanisms of IPL.

## 1. Introduction

Dry eye disease (DED) is a multifactorial disease of the ocular surface that is characterized by a loss of homeostasis of the tear film and is accompanied by ocular symptoms, in which tear film instability and hyperosmolarity, ocular surface inflammation and damage, and neurosensory abnormalities all play etiological roles [1]. The ocular surface symptoms include dryness, pain, burning sensation, foreign body sensation, and blurred vision. Meibomian gland dysfunction (MGD) accounts for most cases of DED, which is caused by partial or diffuse terminal duct obstruction [2]. Treatment for MGD depends on the clinical symptoms and signs. Common therapies include eyelid warming, artificial lubricants, topical azithromycin, and oral tetracycline [3]. However, the efficacy of the treatments could not maintain for a long time.

Intense pulsed light (IPL) is a broad spectrum, noncoherent light. After filtering, the wavelength of IPL is mostly between 500–1200 nm. IPL was first adopted in dermatologic diseases such as facial rosacea, telangiectasia, and pigmented lesions [4]. In 2002, it was found incidentally that dry eye symptoms significantly improved in patients with rosacea treated with IPL [5]. Since then, several studies have investigated the effect of IPL on treating MGD [6–20] and demonstrated a significant improvement in both symptoms and signs.

The recognized mechanisms for IPL treatment of MGD include the following. (1) Temperature around the treatment area rises during IPL treatment, and it can promote the secretion of meibum. By improving the secretion of meibum, the tear film will be more stable, thereby improving the symptoms of MGD. (2) IPL can destroy the telangiectasia in the eyelid margins of MGD patients, thereby reducing inflammatory factors in eyelid tissues. (3) IPL can reduce bacteria on the eyelid skin surface, which are a cause of blepharitis and meibomian gland dysfunction [8, 21]. Recently, the in vivo confocal microscope (IVCM) was widely used, which enables corneal visualization and evaluation in vivo [22]. There are some studies that investigated the corneal nerve changes in DED with IVCM, and these studies observed an obvious decrease in corneal nerve length or density [23–25]. However, the corneal nerve changes remained unknown following IPL treatment.

The present research applied IPL combined with meibomian gland expression in treating MGD and compared the associated ocular surface symptoms, signs, and other parameters following IPL therapy, especially corneal nerve changes.

## 2. Materials and Methods

### 2.1. Patients

This prospective study adhered to the tenets of the Declaration of Helsinki and was approved by the Human Research and Ethics Committee of Peking University Third Hospital. The study was registered at the Chinese Clinical Trial Registry under the registration number ChiCTR1900020576. Informed consent was obtained from each of the participants.

Patients were recruited from the Department of Ophthalmology at Peking University Third Hospital between November 2018 and July 2019. The inclusion criteria for this study were as follows: (1) age >18 years; (2) Fitzpatrick skin types I–IV; (3) at least one symptom including dryness, pain, burning sensation, foreign body sensation, or blurred vision as well as an Ocular Surface Disease Index (OSDI) > 12; (4) clinical signs of meibomian expressibility ≥1 or ocular surface staining; (5) willingness to comply with the treatment and follow-up schedule in this study. Exclusion criteria included the following: (1) acute inflammation; (2) previous ocular surgery or trauma; (3) abnormal eyelids, such as eyelid closure insufficiency, entropion or ectropion; (4) pregnancy and/or nursing; (5) systemic immune-related diseases, such as Sjogren's syndrome, Stevens–Johnson syndrome, and rheumatism; (6) the presence of active skin lesions, skin cancer, or other specific skin pathology; (7) dry eye physiotherapy or anti-inflammation drugs within 1 month; (8) other ophthalmic diseases or conditions judged by the researcher as unsuitable for this clinical trial.

### 2.2. IPL Treatment and Meibomian Gland Expression (MGX)

All IPL treatments were completed using an IPL machine (RH-1; Ruihao, Shanxi, China). Each patient in our study underwent three consecutive treatments at an interval of 3-4 weeks, and follow-up was completed 4 weeks after the last treatment.

**Figure 1:**
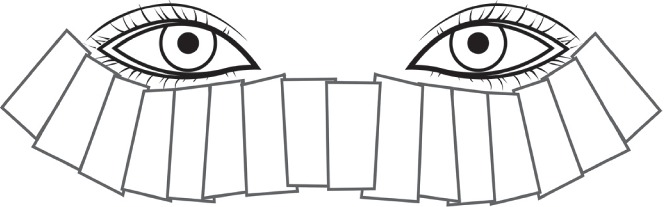
Treatment area of the eyelids: the treatment areas are the lower eyelids. Each rectangle represents a single IPL pulse.

The energy was determined from 11–14 J/cm^2^ according to the skin type. A mode comprising a wavelength of 560 nm–1200 nm and 3 pulses was applied in our treatment. IPL-Aid Eye Shields were placed on the eyes, and we applied a cooling gel to the treated area. Approximately 16 overlapping pulses were processed: 8 pulses on each side on the area below the lower eyelid, as shown in Figure 1. The opposite side was the same. The same procedures were repeated one more time. MGX was processed after IPL treatment. Meibomian gland expressor forceps were used to squeeze meibum from the upper and lower meibomian glands. The IPL therapy and MGX were performed by a single investigator (ZYL).

### 2.3. Clinical Assessment

The clinical assessments of the enrolled patients were conducted in the following order and the influence of a preceding test on the subsequent test was minimized: OSDI questionnaire, inferior tear meniscus height (TMH), tear break-up time (TBUT), slit-lamp examinations, and in vivo confocal microscopy (IVCM). An interval of 5 minutes was required between different tests. All of them were performed before the therapy as a baseline examination, and we performed a follow-up examination at 3-4 weeks after the last treatment.

#### 2.3.1. Ocular Surface Disease Index (OSDI)

We evaluated subjective symptoms through the OSDI questionnaire, which was a scale ranging from 0 to 100. The patients needed to answer 12 questions and give a score of 0 to 4 to these items. OSDI = (sum of scores for all questions answered × 100)/(total number of answered questions × 4).

#### 2.3.2. Tear Break-Up Time (TBUT) and Tear Meniscus Height (TMH)

TBUT and TMH were estimated using a noninvasive ocular analyzer, the Keratograph 5M (OCULUS, Wetzlar, Germany). A previous study had proven the repeatability and reproducibility of keratography measurements [26]. The analyzer captured an infrared photograph of the anterior segment of eyes enabling assessments of TBUT and inferior TMH. Patients were asked to blink several times and then keep their eyes open as long as possible. We measured the first break-up time and the average break-up time of the tear film. TMH was defined as the length of a vertical line extending from the top of the inferior tear meniscus to the eyelid margin, which could be measured by the Keratograph 5M as well.

#### 2.3.3. Slit-Lamp Examinations

Slit-lamp examinations included evaluations of eyelid margin signs, meibomian gland assessments, and corneal fluorescein staining. Eyelid margin signs were evaluated as follows: rounding of the posterior margin, irregularity of the margin, lash loss, trichiasis, and telangiectasia. Fixed pressure was applied to the meibomian glands to evaluate the meibum quality and expressibility for each gland. Meibum quality was scored as 0, clear; 1, cloudy fluid; 2, cloudy particulate fluid; or 3, inspissated like toothpaste. Meibum expressibility was scored as 0, all glands expressible; 1, 3-4 glands expressible; 2, 1-2 glands expressible; or 3, no glands expressible. Three positions (nasal, central, and temporal) of the upper or lower eyelid and 5 glands at each position were evaluated, and the score was calculated as the sum of 3 positions. The maximum score was 9, and a score of 3 or above was considered abnormal. The standard was performed according to the previous reports [27]. Corneal staining was assessed by applying a drop of fluorescein sodium and then was viewed by slit-lamp instrumentation using cobalt blue illumination. We divided the cornea into 4 quadrants. Staining was scored from 0 to 3 in each quadrant and summed.

#### 2.3.4. In Vivo Confocal Microscopy (IVCM)

IVCM (Heidelberg Engineering, Heidelberg, Germany) is a noninvasive imaging modality that enables the observation of different layers of the cornea in vivo at the cellular level. A drop of oxybuprocaine was administered into the conjunctiva sac for topical anesthesia, and a drop of hydroxypropyl methylcellulose was placed on the tip of the TomoCap to improve the image quality. Participants were instructed to fixate on a near target, and we moved the machine forward so that the IVCM could examine the corneal region. A total of 30 to 40 pictures were captured for each eye. The images with artifacts or unclear images were excluded. For each cornea, images were selected, which has the richest plexus. An experienced investigator screened and captured the pictures and chose 3 to 5 images. Quantification of the corneal nerves was performed on all of these images by another investigator. All ophthalmologic examinations were performed by two investigators (RXT and WSS).

## 3. Statistical Analysis

**Figure 2:**
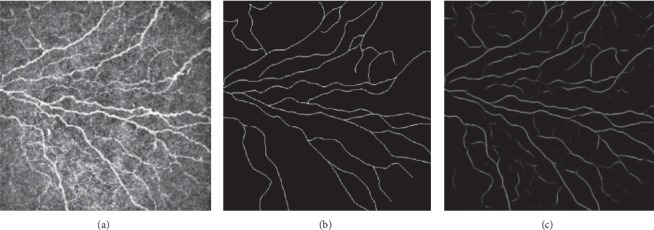
An example of corneal nerve annotation. (a) The original image of a participant in our study. (b) Corneal nerves identified by manual annotation. (c) Corneal nerves identified by the deep learning model.

SPSS software version 22 (SPSS, Inc., Chicago, IL, USA) was used for statistical analysis. The descriptive data are presented as the mean and standard deviation (SD). The mixed linear model was used to compare the clinical assessment parameters before and after treatment. *p* values less than 0.05 were considered statistically significant. We applied a pretrained deep learning model to analyze the corneal nerves, which allowed automatic nerve detection and segmentation and calculation of corneal nerve fiber length. The deep learning model was established based on a U-net architecture pretrained with more than 5000 corneal nerve images examined by IVCM. It achieved an AUC of 0.96 and a sensitivity of 96% (not shown in the present research). We chose 3 to 5 images of each eye and obtained the average corneal nerve fiber length (CNFL).  Figure 2 shows the corneal nerve image taken by IVCM (a), manual annotation (b), and annotation of the artificial intelligence model (c).

## 4. Results

### 4.1. Demography

Fifty-three patients, including 8 males and 45 females, were enrolled in our study. The mean age was 52.94 ± 17.94 years.

### 4.2. Clinical Symptoms and Signs

As shown in Table 1, the OSDI score was significantly reduced after the IPL treatment compared with the baseline (*P* < 0.001). There was no significant change in the average TBUT. The baseline TMH was 0.18 ± 0.01 cm, and after IPL therapy, it reached 0.20 ± 0.07 cm, which was also a significant improvement (*P*=0.003). Furthermore, meibomian gland assessment values (including meibum quality and expressibility) were significantly improved after treatment (*P*=0.001, respectively). Eyelid margin abnormalities, including telangiectasia and irregular and corneal staining, were significantly improved, as shown by the decrease in their scores.

### 4.3. Corneal Nerves and IPL Treatment

The IVCM was applied to assess the effect of IPL treatment on corneal nerves. As shown in Table 2, CNFL was 1.82 ± 0.68 mm before the treatment and 2.45 ± 0.65 after the treatment, and this improvement was statistically significant (*P* < 0.001). The artificial intelligence model provided a concrete length and the pixel value.

## 5. Discussion

MGD is a highly prevalent disease. However, efficient therapy remains to be clarified. IPL treatment in MGD was first reported in 2016 [10] and showed excellent performance. There are many proposed hypotheses of IPL treatment mechanisms, although there is no strong evidence to support some of these possible mechanisms. Our study aimed to investigate the efficiency of IPL treatment and explore the mechanism involved in IPL treatment of MGD.

To the best of our knowledge, we are the first to show corneal nerve alterations after IPL treatment. Moreover, an artificial intelligence model was applied to analyze the corneal nerves quantitatively. Symptoms (measured by OSDI) and signs (TMH, meibomian gland expressibility, meibum quality, eyelid margin abnormalities, and corneal staining) as well as corneal nerve condition after IPL treatment were evaluated in our investigation.

The OSDI has been reported to be a reliable and valid parameter for discriminating the severity of dry eye diseases [28, 29]. We found that the value was significantly decreased after IPL treatment compared with baseline, which is in agreement with other investigators' studies [13, 14]. Self-reported symptoms shown by the Standard Patient Evaluation of Eye Dryness (SPEED) score were also reduced (indicating reduced symptoms) after IPL treatment in previous studies [6, 11, 20, 30]. Based on these results, we concluded that IPL could relieve symptoms to a certain degree.

Eyelid warming is effective in relieving symptoms to some extent, although this effect is transient. A mathematical model explains the selective photothermolysis of IPL, concluding that the light energy is absorbed by hemoglobin and that the temperature in the vessel is increased. In small vessels (100 *µ*m), the temperature ranges from 40°C to 70°C, and this temperature cannot coagulate the vessel; however, it might be above the phase-transition temperature (the temperature at which meibum switches into another phase). Under the effect of IPL, the meibum is heated and liquefied, which may improve the expression of the meibum from the inspissated glands. In our study, improvement of the meibomian gland, factors such as meibum quality and expressibility, verified that the possible mechanism of IPL treatment is that it may increase the local temperature, promoting melting and liquefying of the meibum.

Eyelid margin abnormalities (telangiectasia and irregularity) were evaluated in our study. They were all ameliorated after IPL therapy, similar to previous studies [10, 18, 20]. Selective photothermolysis elevates temperature and coagulates blood vessels when it exceeds 70°C, and it is the main mechanism for which IPL is applied to vascular disorders in dermatology [31, 32]. It has also been reported that in larger vessels (150 *µ*m), the temperature could rise to 80°C–90°C and cause the destruction of blood vessels. By coagulating the vessels, the inflammatory factors released from abnormal vessels decreased, subsequently improving the clinical presentation of MGD.

There are many proposed mechanisms through which IPL treats MGD, including (but not limited to) thrombosis of abnormal blood vessels, liquefaction of the meibum, reduction of inflammatory mediators, and photomodulation. To elucidate more mechanisms by which IPL treats MGD, we conducted IVCM examinations and compared the corneal nerves after treatment. IVCM provides a continuous and dynamic observation of corneal anatomy, which helps us clarify the possible mechanism of IPL treatment for MGD. In our study, the CNFL increased with statistical significance following IPL compared to the baseline. Previous research demonstrated that innervated corneal tissue is one of the most powerful pain generators in the body [33]. Destruction of the corneal nerve might cause perceptions such as burning, stinging, and pain. Jiang et al.'s [18] study indicated that corneal nerve injury can increase neurogenic sensitivity through proinflammatory factors [18, 34]. Previous research has studied the pathophysiology of corneal nerve injury and concluded that corneal nerve injury could lead to acute axonal injury, which can result in a reduced threshold potential of ion channels in corneal nerve endings because of the release in inflammatory mediators such as substance *P*, tumor necrosis factor-*α*, and interleukin-1 [33]. Photomodulation has the ability to modulate cellular metabolism through the absorption of photons by photoreceptors, including cytochrome c oxidase in mitochondria and calcium ion channels [35]. Anti-inflammation effects including pain relief, healing stimulation, and reduction of inflammation factors are a hallmark of photomodulation [36]. Clinical applications of photomodulation for inflammation included achilles tendinopathy, thyroiditis, muscles, psoriasis, arthritis, and alopecia areata [37–42]. Reduced inflammation and attenuation of reactive oxygen species (ROS) could promote corneal nerve promotion [43]. Previous studies have also reported the function of low-level lasers in promoting regenerative processes in injured nerves, which might be associated with photomodulation [39, 44, 45]. Therefore, neurotrophism might be one of the mechanisms in IPL treating MGD. However, the eye shield was applied during IPL treatment, which blocked the cornea contacting most of the light, that alleviated the direct effect of IPL on cornea. Thus, indirect efficacy of IPL in improving corneal nerve should not be neglected, including the less inflammation and better ocular surface microenvironments, and further research is required to further clarify the specific mechanisms.

We applied a fully automated CNFL analysis in our study, and the average time for analyzing each image was much shorter than that incurred by manual work. In our previous investigation, we compared the results of artificial intelligence and manual work, which showed excellent performance of the former. This automated nerve-tracing model detected the corneal nerves and calculated the results of CNFL. We obtained better performance than that of the semiautomated software, and our system is much faster than manual annotation.

However, there are some limitations to our study. (1) First, in our study, MGX was performed after IPL treatment, as was done in other [9, 14] studies. We lacked a control group because MGX itself may exert a role in improving MGD symptoms and meibomian gland function. Further studies should be conducted with placebo controls. (2) The follow-up time was limited to 4 weeks, and we would need further observation to evaluate the long-term effectiveness of IPL treatment. (3) The meibomian glands of the upper eyelids cannot be treated directly because IPL might injure the underlying ocular structures. (4) The fourth limitation was the standard of patients included. According to the International Workshop on Meibomian Gland Dysfunction, MGD is categorized into 5 levels. The patients we included were from level 3 to level 5 (symptomatic mild, moderate, and severe), and we included only patients whose OSDI was over 12 [27].

## 6. Conclusion

Meibomian gland dysfunction (MGD) is the most common cause of dry eye, which results in alteration of the tear film, including symptoms such as dryness, pain, burning sensation, foreign body sensation, and blurred vision. The results of our study showed an improvement in symptoms, signs, and corneal nerve condition after IPL combined MGX treatment. According to the IVCM results regarding the corneal nerves, we hypothesize that the neurotrophism might be one of the mechanisms in IPL therapy. Further studies are needed to explore the long-term effectiveness and safety.

## Figures and Tables

**Table 1:** Clinical assessments of pre- and posttreatment.

	Pretreatment	Posttreatment	*P*
OSDI (score)	36.07 ± 1.67	30.04 ± 1.93	<0.001^*∗*^
TBUT (s)	9.58 ± 3.04	7.52 ± 0.80	0.385
TMH (cm)	0.18 ± 0.01	0.20 ± 0.07	0.003^*∗*^
Meibomian gland assessments				
Secretion quality (0–3)	1.98 ± 0.80	1.61 ± 0.89	0.001^*∗*^
Expressibility (upper, 0–9)	6.73 ± 2.09	5.63 ± 2.28	0.001^*∗*^
Expressibility (lower, 0–9)	7.12 ± 2.10	5.74 ± 2.36	0.001^*∗*^
Eyelid margin abnormalities			
Telangiectasia (0/1)	0.70 ± 0.46	0.50 ± 0.50	0.001^*∗*^
Irregular (0/1)	0.81 ± 0.90	0.72 ± 0.45	0.030^*∗*^
Corneal staining (score)	0.77 ± 2.54	0.30 ± 2.38	0.022^*∗*^

*p* values of less than 0.05 were considered statistically significant and are expressed as ^∗^.

**Table 2:** Comparison of corneal nerves before and after treatment.

	Pretreatment	Posttreatment	*P*
Corneal nerve			
CNFL (mm)	1.82 ± 0.68	2.45 ± 0.65	<0.001^*∗*^
CNFL (pixel)	1898.53 ± 684.83	2592.03 ± 675.72	<0.001^*∗*^

CNFL is defined as the total length of all nerve fibers visible in the IVCM image per square millimeter. *p* values of less than 0.05 were considered statistically significant and are expressed as ^∗^.

## Data Availability

The data, models, or code used to support the findings of this study are available from the corresponding author upon request.
